# Solid tissue simulating phantoms having absorption at 970 nm for diffuse optics

**DOI:** 10.1117/1.JBO.22.7.076013

**Published:** 2017-07-20

**Authors:** Gordon T. Kennedy, Griffin R. Lentsch, Brandon Trieu, Adrien Ponticorvo, Rolf B. Saager, Anthony J. Durkin

**Affiliations:** a University of California, Beckman Laser Institute and Medical Clinic, Irvine, California, United States; b University of California, Department of Biomedical Engineering, Irvine, California, United States

**Keywords:** tissue simulating phantoms, diffuse optical spectroscopy, turbid media, inverse adding-doubling, integrating sphere, water

## Abstract

Tissue simulating phantoms can provide a valuable platform for quantitative evaluation of the performance of diffuse optical devices. While solid phantoms have been developed for applications related to characterizing exogenous fluorescence and intrinsic chromophores such as hemoglobin and melanin, we report the development of a poly(dimethylsiloxane) (PDMS) tissue phantom that mimics the spectral characteristics of tissue water. We have developed these phantoms to mimic different water fractions in tissue, with the purpose of testing new devices within the context of clinical applications such as burn wound triage. Compared to liquid phantoms, cured PDMS phantoms are easier to transport and use and have a longer usable life than gelatin-based phantoms. As silicone is hydrophobic, 9606 dye was used to mimic the optical absorption feature of water in the vicinity of 970 nm. Scattering properties are determined by adding titanium dioxide, which yields a wavelength-dependent scattering coefficient similar to that observed in tissue in the near-infrared. Phantom properties were characterized and validated using the techniques of inverse adding-doubling and spatial frequency domain imaging. Results presented here demonstrate that we can fabricate solid phantoms that can be used to simulate different water fractions.

## 1 Introduction

Biomedical diffuse optical imaging systems require tissue phantoms that mimic the optical properties of tissue for their development, characterization, and calibration. Many different phantom systems have been investigated using a variety of host matrices, scattering particles, and absorbers. For an excellent review, the reader is directed to Pogue and Patterson^1^ and references therein.

When used for calibration or the validation of models, it is desirable that the optical properties of the phantom are similar to those of the target tissue to reduce the likelihood of measurement error related to interpolation and extrapolation of optical properties. Additionally, for wide-field imaging, homogeneity over a large area may also be required. For some applications, it is sufficient to reproduce the reduced scattering and absorption properties at discrete wavelengths; however, for spectroscopic measurements, it may also be necessary to approximate the spectra of the optical properties of tissue parameters over the range that they are measured.

**Fig. 1:**
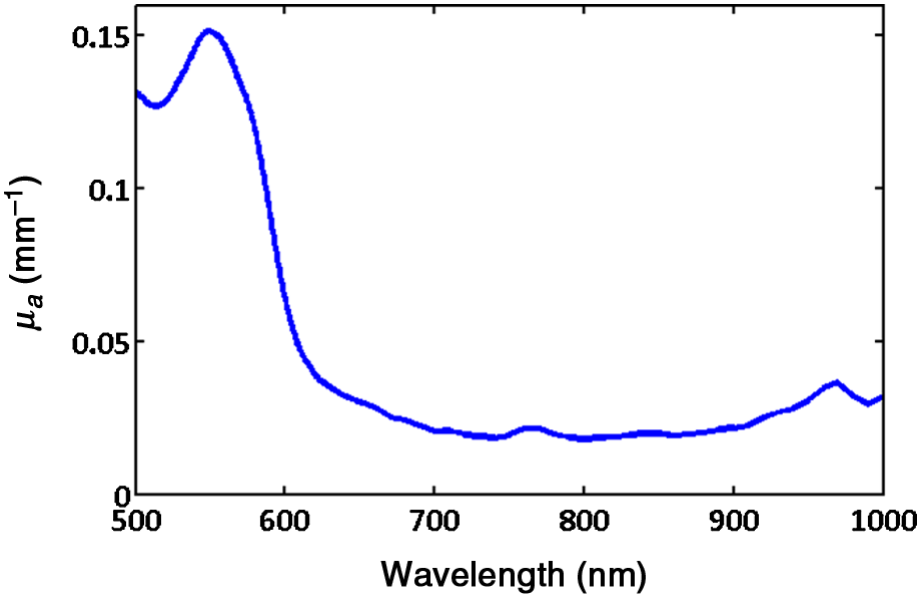
Absorption spectra of forearm.

A typical absorption spectrum obtained from spatial frequency domain measurements^2^^,^^3^ of skin (human forearm) from 500 to 1000 nm is presented in Fig. 1. For superficial tissues, such as skin, phantom fabrication efforts have focused primarily on mimicking the absorption characteristics associated with oxy- and deoxy-hemoglobin which are featured in the 500- to 600-nm range. Biomedical problems concerning monitoring edema within the context of burn wounds, inflammation due to infection or foreign body response, or simple cosmetic hydration measurements are likely to benefit from new spectral imaging technologies; however, the means for systematically varying the absorption spectrum at 970 nm to simulate varying water fraction in a solid phantom in order to test and validate these devices have been difficult to come by.

Water containing phantoms based on fat emulsions have been widely used. In particular, Intralipid^®^ (Fresenius Kabi AB. Uppsala, Sweden) diluted with water has been used extensively,^4^^–^^6^ and significant work has been carried out to determine properties and stability of these phantoms and reliable methods of producing them.^7^^–^^9^ It is relatively straightforward to add other chromophores such as blood in order to obtain realistic absorption spectra.^10^ Unfortunately, these phantoms have a limited shelf life (hours to days). The primary role of the lipid droplets in these phantoms is to provide the scattering. To obtain physiologically relevant reduced scattering coefficients, only a few percent Intralipid^®^ is required, therefore, these phantoms typically contain greater than 95% water. While the water fraction can be adjusted by changing the fat to water ratio, the scattering provided by the fat droplets is proportional to their number density. Decreasing the water fraction increases the fat content thus increasing the scattering.

Merritt et al.^11^ reported variation of lipid and water content in fat emulsion phantoms; however, the scattering properties of the emulsions were not disclosed. Subsequently Quarto et al.^12^ investigated three phantom types that enabled the relative fractions of fat and water to be varied, including the emulsion system described by Merritt et al. They reported that for this recipe the reduced scattering was exceptionally high. It could be reduced somewhat by lowering the amount of the emulsifying agent but at the cost of reduced stability; the phantoms would only last a few hours before separating.

Agar/gelatin phantoms do offer the potential of controlling the water fraction and can be molded into three-dimensional (3-D) shapes but again suffer from limited lifespan of a few weeks/months.^13^^,^^14^ Phantoms constructed using these materials also have fragile mechanical properties and require refrigeration to maintain optical properties and minimize evaporation of the water. Because these phantoms are not particularly robust and require very gentle handling and storage, their usefulness within a clinical context is somewhat limited.

Michaelsen et al.^15^ have reported a semisolid animal fat-based breast phantom that is capable of reproducing physiological properties where the water content can be varied over a wide range. These phantoms also require refrigeration and showed stable optical properties for several weeks.

Solid phantoms fabricated from materials such as epoxy,^16^ polyurethane,^17^ and silicone^18^^–^^23^ are more durable, easy to handle and transport and can exhibit stable optical properties for periods extending to several years. This makes them popular for calibration and routine testing of instrumentation. Additionally, they can be molded, machined, or 3-D-printed^24^^–^^27^ into complex 3-D shapes having homogeneous, heterogeneous, and layered optical properties that are useful for more complex tissue models. However, we are unaware of phantoms containing physiological water fractions in the literature. Room temperature vulcanizing (RTV) poly(dimethylsiloxane) (PDMS) is a compliant material having a refractive index close to tissue. It is inexpensive, readily available and relatively straightforward to mold into 3-D shapes.^18^ Several groups, including our own, have successfully developed phantoms based on PDMS. These have been used for calibrating and validating various diffuse optical spectroscopy and imaging devices over the years for applications related to skin cancer, breast cancer, muscle physiology, and burn wound triage.^21^^–^^23^

The strongly hydrophobic property of silicone makes it difficult to introduce significant amounts of water into phantoms. Since the water fraction of tissue is commonly in the range of 60% to 90%, it is not possible to create silicone phantoms having physiological water content. In this paper, we describe solid silicone tissue phantoms that incorporate a near-infrared pthalocyanine dye to simulate a variation in tissue water absorption. To our knowledge, this dye has not previously been employed in tissue simulating phantoms.

## 2 Materials and Methods

The fabrication of the tissue phantoms follows the method described by Ayers et al.^19^ The base phantom material was RTV silicone rubber (P4, Eager Plastics Chicago, Illinois). Scattering was by provided by adding titanium oxide powder [Titaniun (IV) Oxide, anatase 248576, Sigma Aldrich] and the near-infrared absorption was obtained using the phthalocyanine dye 9606 (Fabricolor Holding Int’l, Paterson, New Jersey).

**Fig. 2:**
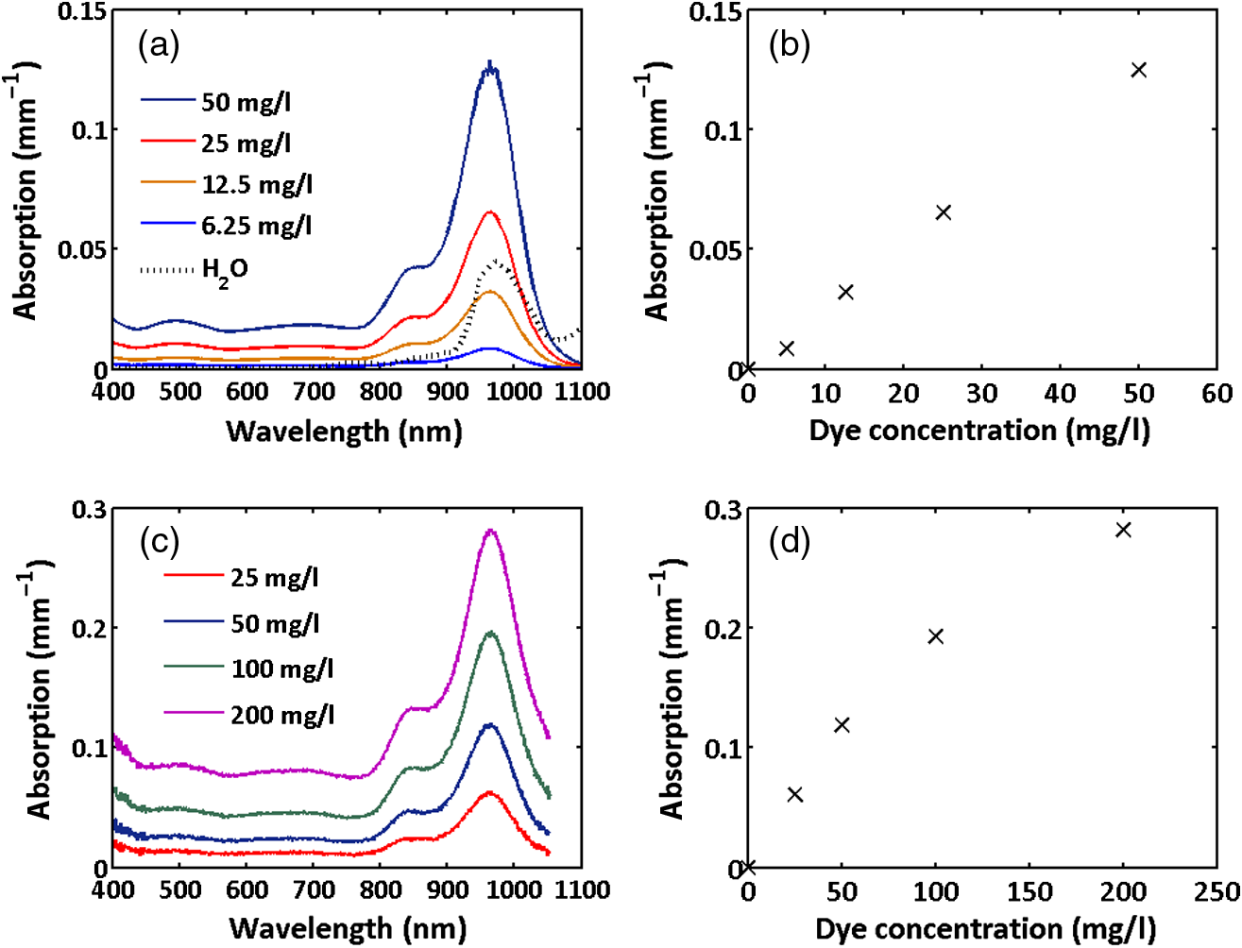
Absorption spectra of the 9606 dye: (a) spectra for the dye dissolved in acetone, (b) variation of absorption at 970 nm with dye concentration in acetone, (c) dye in cured PDMS, (d) variation of absorption at 970 nm with dye concentration in silicone for both hosts the absorption monotonically increases with dye concentration at all wavelengths.

Figure 2(a) shows the absorption spectrum of a cuvette of dye dissolved in acetone measured using a spectrophometer. The dye exhibits a broad absorption from 800 to 1100 nm with peak at 972 nm. The absorption spectrum for water^28^ is also shown for comparison. While the dye absorption is broader than that of water spanning both the water and lipid peaks at 920 and 970 nm, it nevertheless is a reasonable approximation.

The peak absorption at 972 nm as a function of dye concentration in acetone is presented as Fig. 2(b), showing a linear variation of absorption coefficient with concentration. Figures 2(c) and 2(d) show the absorption and variation in peak absorption with concentration for 5-mm-thick cured silicone slabs having no scattering particles measured relative to the same thickness cured silicone with no dye. The spectrum is somewhat broader than the dye in solvent but the peak remains at the same wavelength. The variation of absorption with concentration is somewhat sublinear at higher concentrations.

${\mathrm{TiO}}_{2}$ powder was added to the curing component agent at a concentration of $1\;g\;{\mathrm{kg}}^{-1}$, and the mixture was sonicated for 3 h, mixing regularly to break up any clumps and disperse the powder evenly. Previous experience indicated that this results in a reduced scattering coefficient of $∼1\;{\mathrm{mm}}^{-1}$ at a wavelength of 700 nm.

A stock solution of dye was made by dissolving dye powder in acetone at a concentration of $10\;\mathrm{mg}\;{\mathrm{ml}}^{-1}$ and was added to the base component in amounts ranging from 0.85 to $3.4\;\mathrm{ml}\;{\mathrm{kg}}^{-1}$ and vigorously mixed using an electric drill with a mixing attachment. The two components were then combined and mixed using the electric drill for ∼2  min. The mixture was poured into a 100×100  mm square mold whose base was lined with 320 grit sandpaper to reduce specular reflection from the sample surface. Three-hundred grams of mixture resulted in ∼30-mm-thick phantoms. The molds were placed in a vacuum chamber and degassed using a rotary pump at a pressure of 30 to 60 mbar for ∼40  min. Thin slabs (1 to 3 mm) of the material were made at the same time, by removing a few milliliters of the degassed mixture using a syringe and transferring it to Petri dishes. The samples were placed on a level surface and allowed to cure for 24 h. The phantoms were then removed from the molds and set aside for ∼1 week to fully cure before characterizing them. These thin phantoms become an essential part of the benchmark measurement process that employs an integrating sphere and inverse adding-doubling (IAD) computation of optical properties.

## 3 Results and Discussion

**Fig. 3:**
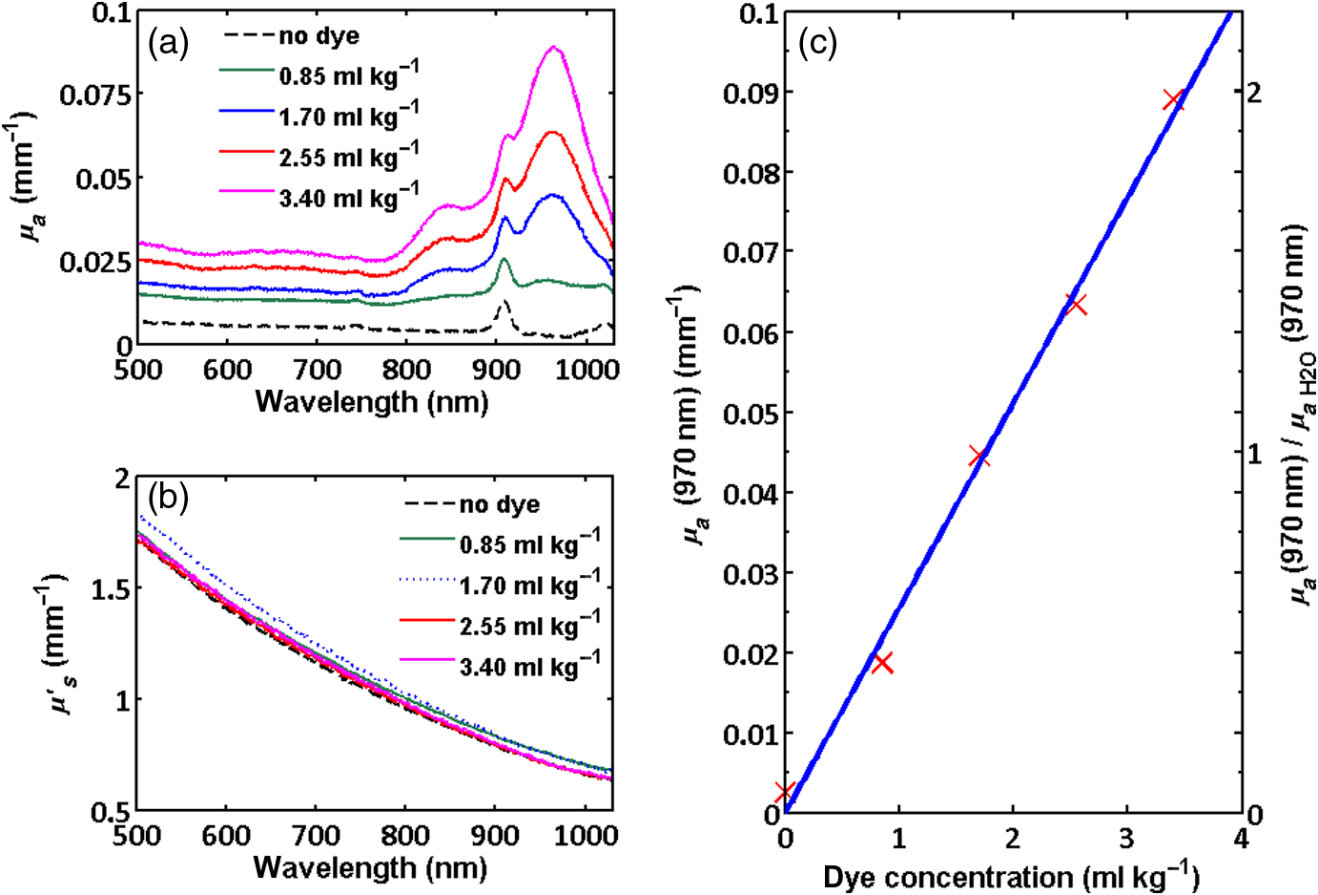
(a) Absorption and (b) reduced scattering spectra obtained using the technique of IAD for thin sheets of phantom having various concentrations of dye. Absorption monotonically increases with dye concentration for all wavelengths. (c) Absorption at 970 nm versus concentration. The left axis shows absolute absorption for the phantoms and the right axis shows absorption at 970 nm normalized to that of pure water.

Measurements of the optical properties of these homogenous phantoms were performed using a variety of techniques and instruments. The thin samples were measured using the technique of IAD.^29^ In our broadband implementation of this technique, the sample was placed either at the entrance port (for transmission measurements) or at the exit port (reflection measurements) of an 8-in. diameter integrating sphere and a collimated beam from a broadband light source having a diameter of ∼6  mm was incident on the sample.^30^ A fiber-coupled cooled CCD spectrometer having a wavelength range of 450 to 1000 nm and resolution of ∼1  nm was used to measure the transmitted and reflected spectra relative to a diffuse reflection standard (Labsphere SRS-99-020, 99%). These data were processed using MATLAB code incorporating the IAD code written by Scott Prahl^31^ to obtain the reduced scattering and absorption spectra. Additional details of the measurement system can be found in Burns et al.^30^ Figure 3 shows the calculated reduced scattering and absorption spectra for phantoms containing stock dye concentrations of 0.85, 1.70, 2.55, and $3.40\;\mathrm{ml}\;{\mathrm{kg}}^{-1}$. The peak absorption is also plotted as a function of dye concentration.

The absorption spectra are similar to the measurements made using the spectrophotometer. The peak near 915 nm corresponds to an absorption feature associated with the PDMS material. This is not apparent in the spectrophotometer measurements as a matched sample of silicone without dye was placed in the reference arm. For reference, the absorption and scattering spectra of a thin PDMS sample with the same scattering but no 9606 dye is presented. The scattering monotonically decreases with increasing wavelength, as expected. Both the slope and magnitude are very similar for the three samples, indicating that each of the phantoms contained approximately the same number and size distribution of ${\mathrm{TiO}}_{2}$ particles. For the concentrations of dye shown in these measurements, the peak absorption varied linearly with concentration as shown in Fig. 3(c). This should enable the absorption to be easily predicted, although ultimately it is necessary to experimentally measure the optical properties to accurately determine them.

The 3-cm thick phantoms were also measured using the technique of spatial frequency domain imaging (SFDI) that we have described in detail in the literature.^3^^,^^32^ In this wide-field imaging method, sinusoidal patterns having various spatial frequencies are projected onto the sample and the diffusely reflected light is imaged using a camera. The changes in the dc and ac components of the reflected patterns are measured as a function of spatial frequency for each pixel. For a given refractive index and scattering anisotropy, the reduced scattering and absorption coefficients can then be calculated.

Two SFDI instruments were used: (1) a system built in our lab, incorporating broadband illumination and narrowband detection using a camera fitted with a liquid crystal tunable filter^33^ and (2) a commercial system (Oximager RS^™^, Modulated Imaging Inc., Irvine, California) that employs light-emitting diode (LED) illumination and broadband detection.

For the home-built device, measurements were taken at 10-nm intervals from 650 to 1000 nm for incident spatial frequencies varying from 0 to $0.2\;{\mathrm{mm}}^{-1}$. Images were taken with the commercial instrument at center wavelengths of 470, 525, 590, 625, 660, 730, and 850 nm for the same spatial frequencies as the home-built system. For both instruments, a look-up table method described by Cuccia et al.^32^ was used to calculate the reduced scattering and absorption coefficients. Additionally, for the LED-based instrument, the transmission of the digital micromirror (DMD) image projector system and the sensitivity of the CCD camera were low beyond 900 nm. To maximize signal, an image was taken using planar illumination using a 970-nm LED having a beam path that bypassed the DMD. The 660-, 730-, and 850-nm reduced scattering coefficients were used to fit power law scattering spectra of the form

$$\mu_{s}^{\prime }(\lambda )=A{\left(\frac{\lambda }{\lambda_{o}}\right)}^{-b}.$$

This relationship was then used to extrapolate a value for the scattering at 970 nm. We have described this approach previously by Wilson et al.^34^ The absorption coefficients were then calculated given the planar reflectance and the extrapolated reduced scattering coefficient.

**Fig. 4:**
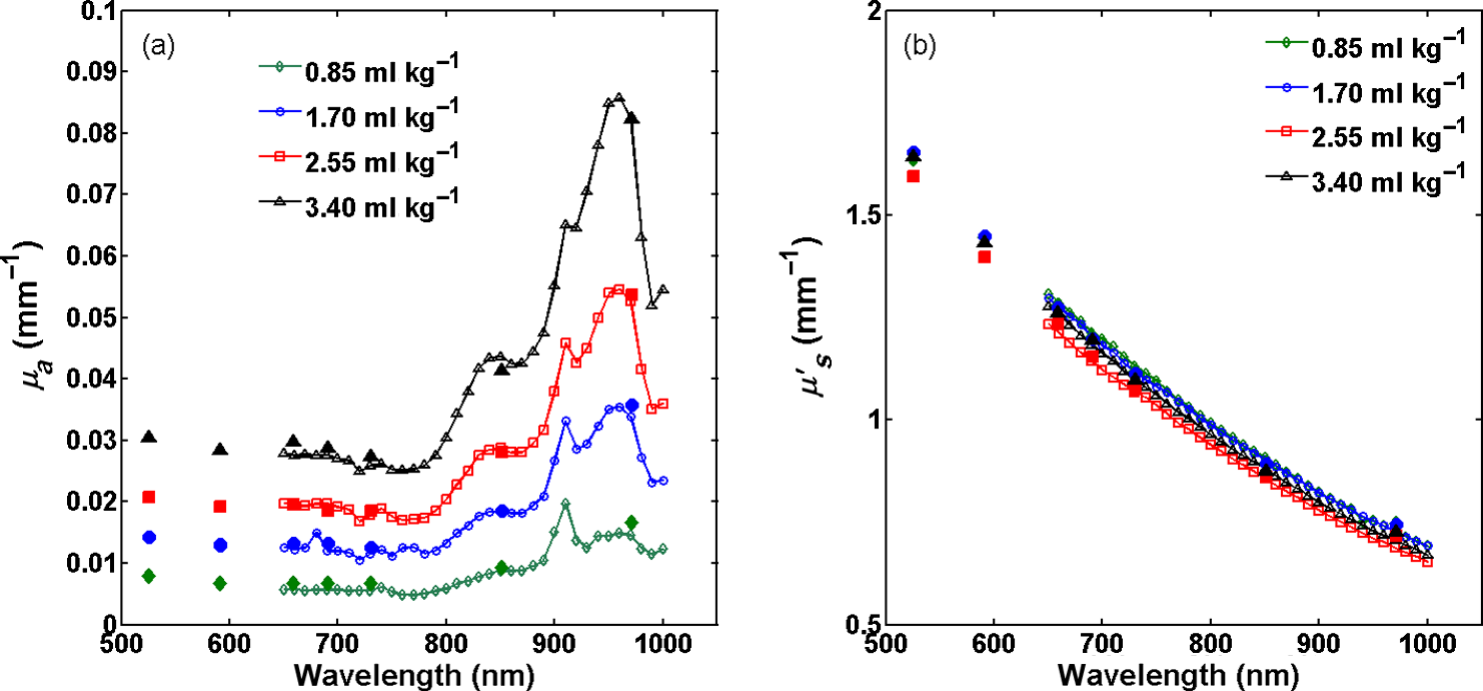
Spectra of (a) absorption and (b) reduced scattering coefficients of 3-cm-thick PDMS phantoms measured by SFDI (central 100×100  pixels). Data from the LED-based system is represented by solid markers and data from the in-house system is presented as line and open markers.

**Fig. 5:**
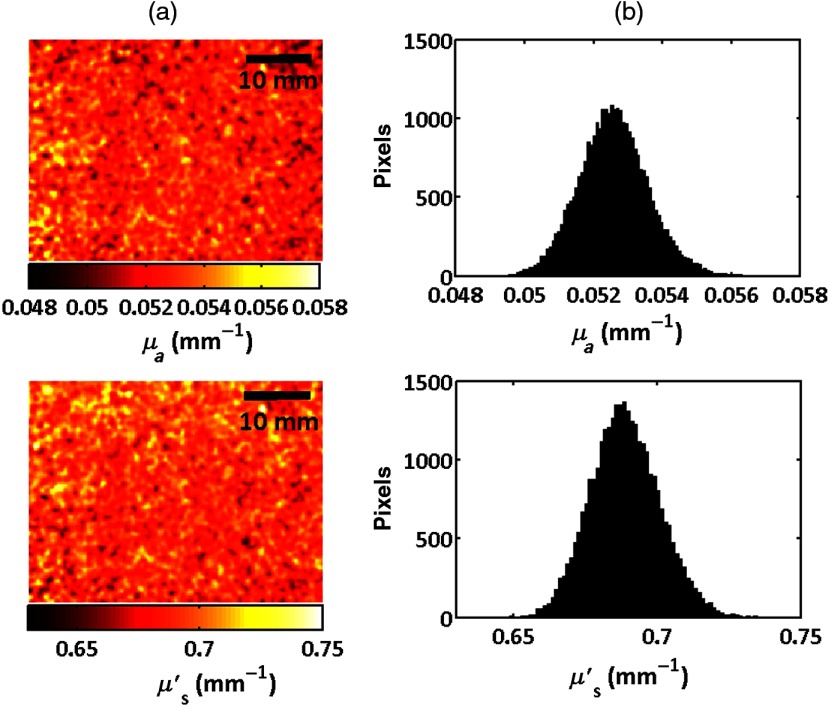
(a) Maps and (b) histograms of the absorption and reduced scattering coefficients at a wavelength of 971 nm for the central 33  mm×45  mm region of a phantom containing 2.55  ml/kg dye.

The absorption and reduced scattering coefficients for the four samples having different dye concentrations measured using the two systems are presented in Fig. 4. Where the wavelength ranges overlap, the measurements are in good agreement with each other and also agree to within 20% of those obtained using the integrating sphere and IAD method that was used with the thin samples. Since SFDI is a wide-field imaging technique, we were able to image the surface of each phantom in order to assess the homogeneity of the optical properties across the sample. Figure 5 shows false color maps of the reduced scattering and absorption coefficients at a wavelength of 970 nm for the central 33×45  mm of the phantom having 2.55  ml/kg dye measured using the in-house system along with the histograms of the optical properties. The size of the image presented in this figure was limited by the field of view of the CCD camera, however, all phantoms exhibited similar uniformity over the central 90% of their surface.

The phantoms showed good uniformity. At a wavelength of 970 nm, the mean reduced scattering coefficient was $0.688\;{\mathrm{mm}}^{-1}$ and the full width at half maximum was $0.022\;{\mathrm{mm}}^{-1}$, or 4% of the mean value for all the samples. For the absorption coefficient, the mean absorption was 0.0138, 0.0335, 0.0525, and $0.0825\;{\mathrm{mm}}^{-1}$ for the phantoms containing 0.085, 1.70, 2.55, and $3.44\;\mathrm{ml}\;{\mathrm{kg}}^{-1}$ dye and the full width at half maxima were 4% 7%, 4%, and 7% of the mean values, respectively.

**Fig. 6:**
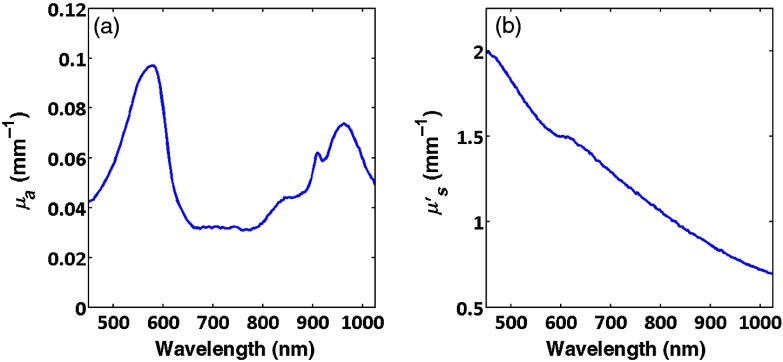
(a) Absorption and (b) reduced scattering coefficients for a phantom containing two dyes; one absorbing in the visible and the other absorbing in the near-infrared.

Finally, Fig. 6 shows the absorption and reduced scattering spectra of a phantom that included the infrared 9606 dye and a dye that has a strong absorption peak in the visible from 450 to 620 nm (dye 5832, Fabricolor Holding Int’l, Paterson New Jersey), in order to simulate aspects of absorption spectral features that are associated with hemoglobin. This illustrates that it is possible to combine dyes to make phantoms having structured absorption.

## 4 Conclusions

We have described the fabrication and measurement of PDMS tissue simulating optical phantoms having independently controllable absorption and scattering. The phantoms incorporate a near-infrared dye to provide absorption that mimics water absorption at 970 nm. While the dye absorption spectrum is not identical to that of water, these phantoms are expected to serve as useful test samples to characterize the ability of diffuse optics systems such as SFDI to determine tissue water fraction. Thin sheets can be fabricated and this dye can be used in conjunction with other dyes enabling the fabrication of phantoms having multiple layered structures and absorption features.
